# Omega-3 Fatty Acids, Furan Fatty Acids, and Hydroxy Fatty Acid Esters: Dietary Bioactive Lipids with Potential Benefits for MAFLD and Liver Health

**DOI:** 10.3390/nu17061031

**Published:** 2025-03-15

**Authors:** Camil Merheb, Sabine Gerbal-Chaloin, François Casas, Mona Diab-Assaf, Martine Daujat-Chavanieu, Christine Feillet-Coudray

**Affiliations:** 1 Institute for Regenerative Medicine and Biotherapy (IRMB), University Montpellier, Institut National de la Santé et de la Recherche Médicale (INSERM), F-34000 Montpellier, France; camil.merheb@inserm.fr (C.M.); sabine.gerbal-chaloin@inserm.fr (S.G.-C.); 2 Dynamique du Muscle et Métabolisme (DMEM), University Montpellier, Institut National de Recherche pour L’agriculture, L’alimentation et L’environnement (INRAE), F-34295 Montpellier, France; francois.casas@inrae.fr (F.C.); christine.coudray@inrae.fr (C.F.-C.); 3 Tumorigenesis Molecular and Anticancer Pharmacology, Faculty of Sciences-II, Lebanese University, Beyrouth 1500, Lebanon; mdiabassaf@ul.edu.lb; 4 Institute for Regenerative Medicine and Biotherapy (IRMB), University Montpellier, Institut National de la Santé et de la Recherche Médicale (INSERM), CHU Montpellier, F-34000 Montpellier, France

**Keywords:** liver, metabolic dysfunction-associated fatty liver disease MAFLD, furan fatty acids, hydroxy fatty acid esters, omega-3 fatty acids, hepatic insulin resistance

## Abstract

Metabolic dysfunction-associated fatty liver disease (MAFLD) is the most common form of chronic liver disease, for which only resmetirom has recently received FDA approval. Prevention is crucial, as it can help manage and potentially reverse the progression of MAFLD to more severe stages. Omega-3 fatty acids, which are a type of polyunsaturated fatty acid (PUFA), have numerous beneficial effects in health and disease, including liver disease. Other bioactive lipids, such as furanic fatty acids (FuFA) and hydroxy fatty acid esters (FAHFA), have also demonstrated several benefits on relevant markers of liver dysfunction in animal and cell models. However, the effects of FAHFAs on hepatic steatosis are inconsistent, and studies on the impact of FuFAs in MAFLD are scarce. Further and more extensive research is required to better understand their role in liver health. The aim of this narrative review is to provide a brief overview of the potential effects of omega-3 fatty acids and other bioactive lipids, such as FuFAs and FAHFAs, on liver disease, with a focus on MAFLD.

## 1. Introduction

The liver is the largest glandular organ in the human body. It performs a variety of vital functions essential for maintaining homeostasis and the proper functioning of various organs. Among its main functions are the metabolism of carbohydrates, proteins, and fats, the detoxification of xenobiotics, and the storage of essential nutrients, vitamins, and minerals. The liver also plays an important role in the immune system. Given its fundamental role in maintaining human health, any impairment of liver function can have serious consequences for the body.

Chronic liver disease is responsible for approximately one million deaths per year [1], as well as a significant reduction in health-related quality of life and increased morbidity and healthcare costs. The main causes of chronic liver disease are chronic hepatitis B virus, chronic hepatitis C virus, alcohol-related liver disease, and non-alcoholic fatty liver disease (NAFLD), recently renamed metabolic dysfunction-associated fatty liver disease (MAFLD) [2]. Chronic liver disease is responsible for approximately one million deaths annually, significantly reducing health-related quality of life and increasing morbidity and healthcare costs. MAFLD accounts for about 50% of chronic liver disease cases and affects approximately 1.7 billion people worldwide [1,3]. A recent meta-analysis revealed an overall prevalence of MAFLD at 30% in the adult population between 1990 and 2019, rising to 38% between 2016 and 2019. This trend suggests an increasing burden of the disease, primarily due to the rising prevalence of obesity and type 2 diabetes (T2D), of which MAFLD is the metabolic liver manifestation. The highest prevalence of MAFLD has been observed in Latin America (44.4%), followed by the Middle East and North Africa (36.5%) [4]. MAFLD encompasses a complex spectrum of liver diseases, ranging from simple steatosis to non-alcoholic steatohepatitis (NASH), recently renamed metabolic dysfunction-associated steatohepatitis (MASH), which can progress to liver fibrosis and cirrhosis, ultimately leading to liver failure and hepatocellular carcinoma (HCC). Between 10% and 30% of MASH patients may develop fibrosis followed by cirrhosis within ten years [5], and 40–60% of cirrhosis patients may develop HCC [6,7]. The precise mechanism of MAFLD remains unclear, but a “multiple hits” hypothesis has been proposed to explain its development and progression [8,9]. In summary, factors such as obesity, poor dietary habits (like a Western diet high in saturated fats and fructose), and genetic risk factors may lead to the development of insulin resistance in skeletal muscle, followed by hepatic insulin resistance. This results in increased hepatic de novo lipogenesis and resistance to the anti-lipolytic effects of insulin on adipocytes, leading to fat accumulation in the liver, particularly in the form of triacylglycerols (TAG). The dysregulation of lipid metabolism causes hepatocellular lipotoxicity, inducing mitochondrial dysfunction, oxidative stress, the generation of reactive oxygen species (ROS), endoplasmic reticulum stress, and hepatic inflammation. Without treatment, chronic hepatic inflammation can lead to fibrosis and cirrhosis. Rezdiffra™ (resmetirom), a beta-selective agonist of the thyroid hormone receptor, was recently approved by the FDA for the treatment of MASH with moderate to advanced fibrosis without cirrhosis, in conjunction with a healthy diet and exercise [10,11]. Nevertheless, preventive measures remain the cornerstone of MASH management. The international expert consensus highlights dietary strategies for MAFLD prevention, emphasizing a balanced diet with controlled energy intake, increased whole grains, plant-based proteins, fish, low-fat dairy, and colorful fruits and vegetables, while reducing red/processed meats, unhealthy fats, sugars, and alcohol. Recommendations also include the Mediterranean/DASH diets, regular physical activity, and minimizing sedentary behavior [12].

This review explores the role of dietary fatty acids in preventing and treating MAFLD and related conditions. It highlights omega-3 polyunsaturated fatty acids (PUFAs), alongside two emerging bioactive lipids with promising health benefits: furan fatty acids (FuFAs) and fatty acid esters of hydroxy fatty acids (FAHFAs).

## 2. Methodology

**Figure 1:**
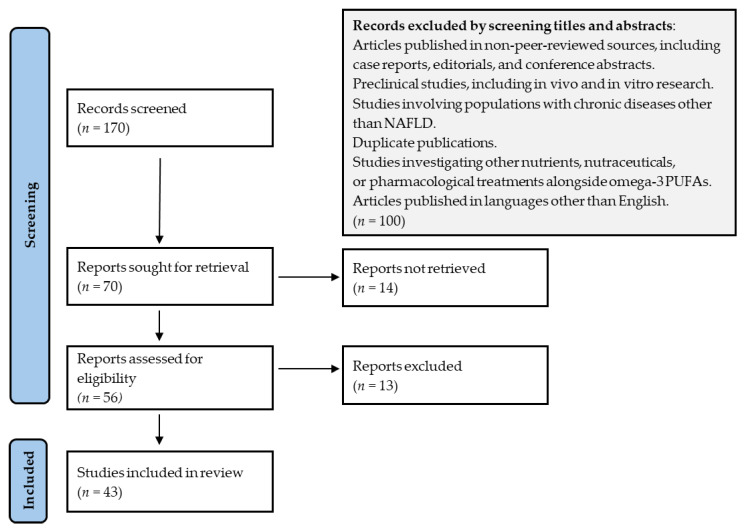
Flow diagram of the eligible papers for omega-3 and NAFLD study.

The bibliographic search was conducted using the PubMed and Web of Science databases. The keywords used were ‘fatty acid esters of hydroxy fatty acids’ and ‘furan fatty acids’, with no restrictions on publication date. The search yielded 124 papers on ‘fatty acid esters of hydroxy fatty acids’ and 158 papers on ‘furan fatty acids’. Due to the limited number of articles, all abstracts were reviewed, and relevant articles were selected based on the content and fully analyzed. In the context of omega-3 fatty acids, a search was conducted using the keywords (“omega-3 PUFA” OR “omega-3 fatty acids” OR “EPA” OR “eicosapentaenoic acid” OR “DHA” OR “docosahexaenoic acid”) AND (“NAFLD” OR “MAFLD” OR “Non-alcoholic fatty liver disease” OR “Metabolic dysfunction-associated fatty liver disease” OR “liver steatosis”), focusing on the literature published in English between January 2010 and February 2025. This search yielded a total of 170 papers, from which 43 articles were selected based on their quality, relevance, and content (see Figure 1). Two review authors (CM and CCF) were independently involved throughout the entire process.

## 3. Omega-3 Fatty Acids

Omega-3 fatty acids are PUFAs with more than one carbon–carbon double bond. Their nomenclature is based on the location of the first double bond, starting from the tail. They are essential fats supplied by the diet, found in fish, certain seafood like algae, leafy vegetables, vegetable oils, and nuts. In the human body, these fatty acids are mainly stored in depot fat as TAG or incorporated into phospholipids (PL), the major structural components of all cellular membranes, where they help maintain membrane structure and fluidity [13]. PUFAs can be acquired through dietary intake or synthesized endogenously via enzymatic desaturation, elongation, and peroxisomal β-oxidation. The enzymatic processes responsible for converting the essential precursors, α-linolenic acid (C18:3*n*-3, ALA) and linoleic acid (C18:2*n*-6, LA), into omega-3 and omega-6 PUFAs may vary across different tissues, including the liver, brain, testes, kidneys, heart, and lungs [14,15]. The metabolic pathway of omega-3 PUFAs begins with α-linolenic acid (ALA, C18:3*n*-3), which serves as the precursor for eicosapentaenoic acid (EPA, C20:5*n*-3) and docosahexaenoic acid (DHA, C22:6*n*-3). This process involves a series of enzymatic steps: desaturation by Δ6-desaturase (Δ6D) and Δ5-desaturase (Δ5D), elongation by elongases 2 and 5, and final β-oxidation in peroxisomes to produce DHA [16]. Docosapentaenoic acid (DPA, C22:5*n*-3) acts as an intermediate in this conversion and can either be transformed into DHA or retro-converted into EPA, serving as a metabolic reservoir [17].

EPA and DHA are abundant in marine fish, while ALA is found in land plants (Table 1).

ALA is converted into EPA and DHA by the rate-limiting enzymes elongase and desaturase, followed by β-oxidation to obtain DHA, with a conversion rate of only 2 to 10% [19]. Therefore, consuming fish and fish oil directly supplies EPA and DHA, bypassing the need for conversion. It is important to note that desaturation and elongation reactions are influenced by various factors, including dietary habits and liver oxidative stress. In fact, studies have shown that in the context of MAFLD, there is a reduction in the endogenous synthesis of PUFAs [15]. The recommended daily intake of combined EPA and DHA for adults is 250 to 500 mg, according to the European Food Safety Authority and Anses (Agence nationale de sécurité sanitaire de l’alimentation, de l’environnement et du travail) [20,21]. With industrialization, consuming the appropriate daily dose of omega-3 PUFA has become increasingly challenging. Ideally, the linoleic acid (LA)/ALA ratio should be less than 5 [21]. While the LA/ALA ratio is of little interest when the physiological requirements for LA and ALA are covered, this ratio becomes important in cases of imbalance due to ALA deficiency and/or LA excess, and even more so when associated with EPA and DHA deficiency (Anses rapport d’expertise collective Saisine n° 2006-SA-0359). In fact, as LA and ALA compete for metabolizing enzymes, higher intakes of omega-6 PUFAs (LA) reduce the availability of these enzymes for the omega-3 pathway. Moreover, arachidonic acid, the metabolite of LA, is strongly associated with inflammation; thus, high intakes of LA increase the risk of inflammation-related diseases, particularly heart disease [22]. As LA is the most abundant PUFA in the Western diet, this ratio has increased dramatically in many countries: 10:1 in France [23], 15:1 in the UK and Northern Europe, and 38–50:1 in India [24,25,26]. Marine organisms are the main source of DHA and EPA. Fortunately, strict regulations are in place to control and limit potential contaminants, such as heavy metals and toxins, ensuring that fish oil supplements meet stringent safety standards and guaranteeing consumer protection. However, despite recommendations for EPA and DHA intake, several factors limit their effectiveness: the perceived unpleasant taste of oily fish, dietary preferences that exclude fish and animal products, and cost [27]. Additionally, marine sources of EPA and DHA are depleting due to rising demand [27,28] and climate warming [29], which raises sustainability concerns. As an alternative to oily fish, algae and seed oils from genetically engineered plants that produce EPA and DHA could provide a sustainable source of omega-3 PUFAs in the human diet. Notable successes include the engineering of *Brassica juncea* and *Camelina sativa* to produce DHA and EPA. While both plants showed significant increases in DHA production, EPA levels were lower in *Camelina sativa* [30]. Furthermore, DPA, which constitutes approximately 5% of typical DHA levels in most tissues from different species, is a major dietary long chain omega-3 PUFAs for many red meat consumers, particularly those with low fish intakes [17]. In fact, omega-3 DPA is more abundant in meat than EPA or DHA. Omega-3 DPA may help maintain an optimal omega-6/omega-3 ratio, which is a key indicator of a preventive diet for managing non-communicable diseases [31].

Over the past 30 years, there has been extensive research and growing public awareness about omega-3 and omega-6 fatty acids and their health impact. Omega-3 PUFAs are known for their anti-inflammatory, anti-thrombotic, and anti-arrhythmic properties. The most robust evidence of their benefits pertains to chronic heart conditions, such as arrhythmia and myocardial infarction, which account for over 500,000 deaths annually in the United States [32,33]. More specifically, the impact of omega-3 DPA on lipid parameters linked to cardiovascular disease prevention is among the best studied, particularly its anti-inflammatory effects, cytokine synthesis inhibition, reduction of thrombosis, and prevention of atherosclerosis [34]. Pro-resolving lipid mediators such as resolvins, maresins, and protectins derived from omega-3 DPA play an essential role in resolving inflammation and regulating immune function [17]. The health benefits of omega-3 DPA may be both unique and overlapping with those of EPA and DHA [35].

DHA and EPA supplementation is also critical during pregnancy for the proper development of the fetal brain and retina [19,36]. Several studies have shown that omega-3 PUFAs are beneficial to individuals with hypertension [37,38] and rheumatoid arthritis, where a daily intake of at least 3 g alleviates symptoms such as morning stiffness and joint swelling pain [37,39]. Omega-3 PUFAs also have beneficial effects on health conditions like Alzheimer’s disease [19]. Recent studies highlight their potential beneficial role in managing psychosis, anxiety, obsessive-compulsive disorders, attention deficit hyperactivity disorder [40,41,42], and binge eating disorder [24].

The effect of omega-3 PUFAs on the liver is well documented. They can reduce plasma TAG levels by inhibiting the synthesis of very low density lipoproteins (VLDL) and TAG in the liver [37,43]. In addition, a recent study using a model of colitis induced by dextran sulphate sodium showed that mice with endogenously increased tissue levels of omega-3 PUFAs produced anti-inflammatory oxylipins and exhibited reduced colitis-induced hepatic oxidative stress, suggesting a protective effect on the liver. Indeed, a meta-analysis documented that the prevalence of MASH is four times higher in patients with inflammatory bowel disease than in the general population, highlighting the link between colon inflammation and steatohepatitis [44]. However, the effect of omega-3 PUFAs supplementation on oxidative stress, a key factor in liver inflammation, remains controversial. The potential benefit is mainly attributed to the anti-inflammatory properties of parent PUFAs and their lipid mediator oxylipin derivatives, and the generation of antioxidant enzymes, limiting ROS accumulation and thereby protecting the liver [45].

The impact of PUFAs on MAFLD has been studied extensively. A recent review compiled multiple preclinical studies highlighting the potential benefits of omega-3 fatty acids in disease management. These studies demonstrated that these bioactive lipids reduce blood triglyceride levels in mice, suppress de novo lipogenesis, and enhance mitochondrial fatty acid oxidation. Additionally, they exhibit promising anti-inflammatory and antifibrotic properties [46]. Moreover, various meta-analyses have demonstrated the effectiveness of marine-based omega-3 PUFAs in humans with MAFLD [47,48,49,50,51,52,53,54,55,56]. Table 2 provides a summary of the effects of omega-3 PUFAs on MAFLD based on the findings from several meta-analyses included in this review. The collective evidence suggests that omega-3 PUFAs may represent a promising therapeutic option for the management of MAFLD. This is supported by consistent reductions in circulating liver enzyme levels, with at least one enzyme showing a significant decrease, improvements in hepatic fat accumulation, and favorable changes in blood lipid profiles, particularly in TAG and TC. Although the impact on glycemic control and body composition remains variable across studies, several meta-analyses have reported positive outcomes in these parameters. Plant-based omega-3 PUFAs (ALA) have also shown some efficacy in MAFLD. In fact, a recent meta-analysis demonstrated that plant-based omega-3 fatty acid supplementation improves ALT enzyme biomarkers, triglycerides, body mass index, waist circumference, and weight loss when combined with lifestyle interventions to increase physical activity and follow a calorie-controlled diet [47]. ALA has also been reported to exhibit potent anti-inflammatory properties [57] which could be mediated through selective COX inhibition, in particular, COX-2 [58].

It is generally accepted that DHA and EPA in triglyceride or ethyl ester forms exhibit lower bioavailability compared to their phospholipid-bound counterparts. This may be attributed to the superior absorption and more efficient tissue incorporation of phospholipid-bound fatty acids relative to those in triglyceride form, likely due to differences in blood transport mechanisms and enhanced access to beta-oxidation pathways [59]. Supporting this, a recent meta-analysis demonstrated that krill oil, which contains phospholipid-bound omega-3, provides superior absorption and efficacy at lower doses compared to the triglyceride-bound omega-3 found in fish oil [60]. However, the influence of dietary fat content on the absorption of omega-3 fatty acids cannot be excluded [59].

Furthermore, a recent meta-analysis showed that PUFA supplementation combined with lifestyle changes reduced hepatic steatosis and liver damage in adult/child patients, as evidenced by a significant reduction in lipid profiles, particularly TAG and, to a lesser extent, LDL-cholesterol, a significant reduction in lipid storage products and visceral adiposity index, a marked decrease in liver enzymes (ALT, AST, GGT), and a positive effect on BMI and waist circumference [61]. However, the limitation here is that it is unclear whether the positive effect on hepatic steatosis is solely due to the action of PUFAs or if it is influenced by the adoption of a healthier lifestyle, which may also contribute to the observed improvement in MAFLD. This highlights the need for a placebo plus lifestyle intervention control group.

On a separate note, data from the Global Burden of Disease 2019 suggest that a diet low in omega-3 PUFAs may be a risk factor for MAFLD-related mortality [62,63]. PUFAs may benefit MAFLD by improving obesity and insulin resistance, mainly by modulating gene expression of peroxisome proliferator-activated receptor alpha (PPARα), sterol regulatory element binding protein-1 (SREBP-1), and carbohydrate regulatory element binding protein (ChREBP) [64,65]. Additionally, a recent meta-analysis reported that plant-based omega-3 PUFAs have a positive effect on glycaemia and the homeostasis model assessment of insulin resistance index (HOMA-IR), a strong indicator of insulin resistance [47]. Finally, some studies suggest that PUFA supplementation may reduce inflammation and play an important role in protecting against disease progression, although the underlying anti-inflammatory mechanisms in the liver remain controversial [47,66]. However, to our knowledge, no histological improvement in inflammation or fibrosis has been demonstrated.

In addition to omega-3 PUFAs, our diet contains other bioactive lipids with health benefits. Among bioactive lipids, short-chain fatty acids (SCFAs), medium-chain fatty acids (MCFAs), conjugated linoleic acids (CLAs), furan fatty acids (FuFAs), and branched fatty acid esters of hydroxy fatty acids (FAHFAs) exhibit potential health benefits. SCFAs, such as acetic, propionic, and butyric acids, are microbial metabolites derived from dietary carbohydrate fermentation in the colon. They regulate cholesterol synthesis, reduce inflammation, and support metabolic health, with alterations in gut microbiota affecting SCFA levels linked to conditions like NAFLD [67]. MCFAs have shown promise in reducing liver steatosis and hepatic injury markers [68], while CLAs have been associated with improved lipid profiles [69]. Most studies on these lipids are based on animal models. Emerging bioactive lipids such as furan fatty acids (FuFAs) and fatty acid esters of hydroxy fatty acids (FAHFAs) hold potential for further benefits. These lipids will be the central focus of the next section of this review.

## 4. Furan Fatty Acids (FuFAs)

FuFAs are valuable minor bioactive components of numerous foods. Fish is the main source of FuFAs. However, as previously mentioned, this reliance on fish raises sustainability concerns. One to four percent of the fatty acid content of fish is furanic fatty acid, which is derived from plants and algae [18]. Shellfish, dairy products, butter, wheat, rice, vegetables, fruits and vegetable oils are also significant sources [69,70]. Table 3 provides an overview of various FuFA sources and their respective concentrations. It can be assumed that Mediterranean populations, who consume a large amount of seafood products and fermented dairy products, may have a higher daily intake of FuFAs.

### 4.1. FuFAs Structure and Metabolism

**Figure 2:**
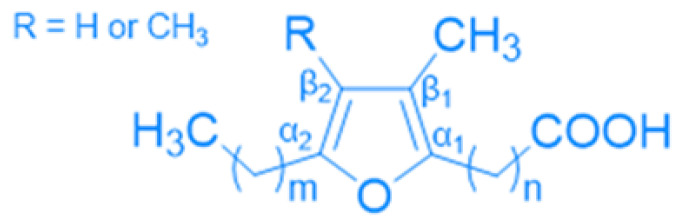
General structure of FuFAs.

FuFAs are naturally occurring fatty acids characterized by a furan ring in the center of the molecule (Figure 2). The molecule carries a carboxyalkyl chain (typical length 9, 11 or 13 carbon atoms) in the α1-position and a propyl or pentyl chain in the α2-position. It also carries either two methyl groups in the β1 and β2-positions or one methyl group in the β1-position and a hydrogen in the β2-position [72]. Discovered in *Exocarpus* seed oil by Morris et al. in 1966 [73], FuFAs were later identified in 1974 in the fish Northern pike (*Esox Lucius*) by Glass et al. [74] and then surprisingly found in 1978 in the latex of *Hevea brasiliensis* [75]. Subsequently, these fatty acids were detected in many other freshwater and marine fish, as well as in plants, algae, and mammals.

There are many nomenclature approaches to FuFAs. The oldest, from 1975, is based on the elution time sequence of the gas chromatography column (F1 to F8) [76]. A more recent nomenclature by Vetter et al. considers the number of carbon atoms in the carboxyalkyl chain, followed by M or D representing methyl or dimethyl in the β-positions, and then a number representing carbon atoms in the α2-position. For example, 11D3 refers to 11-(3,4-dimethyl-5-propyl-2-furanundecanoic acid) [77] (Table 4).

Methylated FuFAs are biosynthesized from PUFAs, particularly LA [78], while unmethylated forms are derived from conjugated linoleic acids [79]. To the best of our knowledge, FuFAs are synthesized only in bacteria, algae and plants. The biosynthesis pathway differs between species and involves lipoxygenase or monooxygenase [18]. In humans, these fatty acids are provided by the diet, though the intestinal microbiota might play a role in their biosynthesis, warranting further investigation. They are mainly present in the liver as cholesterol esters (CE), TAG, and PL, and in the blood as PL or CE [18].

The catabolism of FuFAs also differs between humans and animals, with β-oxidation being the common step in both species. The exact site of catabolism remains uncertain, but some evidence suggests that the liver and intestinal microbiota are two potential sites [72]. Metabolites of FuFAs, known as urofuran acids, are excreted in the urine [80]. 3-carboxy-4-methyl-5-propyl-2-furanopropanoic acid (CMPF) is a major metabolite that has been widely discussed in the literature for its controversial health effects (see below).

### 4.2. Benefits of FuFAs on Liver Health

FuFAs are oxidized fatty acids referred to as oxylipins, a term introduced in 1991 to describe fatty acid-derived oxygenated compounds produced through at least one mono or dioxygenase [81]. These bioactive lipid mediators participate in various physiological processes and can be formed via enzymatic or nonenzymatic free-radical-catalyzed pathways [66].

Antioxidant activity was the first biological property observed in FuFAs. Due to the presence of a furan ring in their structure, FuFAs are considered powerful radical scavengers, protecting PUFAs from rapid oxidation. Lipid oxidation generates reactive lipid species such as peroxyl or alkoxyl radicals. The antioxidant mechanism involves the interaction of these radicals with the furan ring, leading to its opening [82,83]. The level of methyl substitution influences the antioxidant function of FuFAs. Okada et al. demonstrated that dimethylated FuFAs (9D5 and 11D5) significantly inhibited LA oxidation compared to monomethylated FuFA 9M5, while an unmethylated FuFA showed no significant antioxidant activity [84]. These results were confirmed by Batna and Spiteller [85].

More recently, the anti-inflammatory properties of FuFAs have been reported in a few preclinical studies (Table 5). Incubation of 3T3-L1 preadipocytes with FuFA 9M5, alone or with DHA, increased levels of cellular adiponectin, known for its anti-inflammatory properties [86]. Khan et al. found that 11D5 (FuFA F6) induced NETosis in human neutrophils. Neutrophil extracellular traps, consisting of chromatin modified with bactericidal proteins, led to neutrophil activation, defending against pathogens [87]. This suggests that 11D5 may be involved in antimicrobial activity of neutrophils and the attenuation of inflammation. Wakimoto et al. demonstrated in a rat model of adjuvant-induced arthritis that FuFAs from the New Zealand-green-lipped mussel, *Perna canaliculus*, exerted a more potent anti-inflammatory effect than EPA [88].

Regarding their anti-inflammatory and antioxidant properties, FuFAs could play a key role in preventing or combating fatty liver disease, as oxidative stress and inflammation are involved in its development and progression. To our knowledge, we are the only ones to have examined the effects of FuFAs on liver steatosis, highlighting the very limited data on these compounds. Indeed, we recently tested 12 weeks of FuFA supplementation in mice fed a high-fat, high-sugar diet (HFHS) [89]. Given the levels of FuFAs measured in food sources (Table 2), the doses of FuFAs tested were at least 10 times higher than those provided by a typical diet, and chronic supplementation was conducted for 3 months to reflect supranutritional intake. The HFHS diet supplemented with FuFAs limited MAFLD development compared to the HFHS diet alone, as evidenced by lower liver TAG content, lower liver lipid droplet accumulation, and decreased liver expression of Peroxisome Proliferator-Activated Receptor Gamma Coactivator 1 Alpha (PGC-1alpha), a key regulator of gluconeogenesis. FuFAs also demonstrated insulin-sensitizing properties and improved body composition parameters, including decreased fat mass, increased lean and muscle mass, and restored normal energy expenditure. The mechanisms behind FuFAs’ effects on reducing obesogenic diet-induced hepatic steatosis are yet unknown. Their expected antioxidant and anti-inflammatory properties could not be evidenced in the our study, as the HFHS diet alone (without FuFAs) did not induce oxidative stress, and inflammation was not examined in the mice [89]. The reduction in liver fat accumulation may be due to FuFAs’ insulin-sensitizing properties, which needs further investigation.

Plasma CMPF, a major endogenous human metabolite of FuFAs [90], remained unchanged regardless of diet, but this does not exclude CMPF’s involvement in FuFAs’ beneficial effects on steatosis.

CMPF (3-carboxy-4-methyl-5-propyl-2-furanpropionic acid) is indeed a metabolite, and its exact origins from FuFAs are not yet fully elucidated. Current evidence suggests that CMPF is most closely linked to specific furan fatty acids, such as 11D3, which is considered the most likely precursor based on its structural similarity [91]. However, CMPF is not derived from all FuFAs. Furthermore, CMPF might also arise from other metabolic processes, such as the oxidation of polyunsaturated fatty acids (PUFAs), including eicosapentaenoic acid (EPA) [92]. This suggests that CMPF could have multiple precursors beyond just FuFAs and may not be exclusively derived from FuFAs. The findings regarding CMPF on hepatic steatosis are of interest. Clinically, Dai et al. found higher plasma CMPF levels in a Chinese population without MAFLD compared to MAFLD patients, with plasma TAG levels negatively correlated with CMPF. ALT and AST, markers of liver damage, were lower when CMPF was elevated, suggesting CMPF may be a indicative of a healthy liver [93]. Mechanistically, CMPF enters the hepatocyte, mainly via the basolateral organic anion transporter 2 (OAT2/SLC22A7), and inhibits acetyl-CoA carboxylase (ACC), which catalyzes the rate-limiting step of de novo lipogenesis and down-regulates fatty acid β-oxidation in hepatocytes. As a result, an improvement in steatosis was observed in whole livers and isolated primary hepatocytes from male CD1 mice, injected with a preventive dose of CMPF for 7 days, followed by a high-fat diet. Long-term improvement in hepatic lipid accumulation and insulin resistance was observed through a reduction in the Insig2/SREB-1c/FAS pathway involved in de novo lipogenesis, even after 5 weeks of cessation of CMPF injection [94].

Epidemiological studies support a close association between T2D and fatty liver disease, though the causative relation remains unclear. By improving T2D, CMPF may indirectly reduce hepatic steatosis. Lankinen et al. found that CMPF was not associated with impaired glucose metabolism in individuals with features of metabolic syndrome [95], but was associated with impaired glucose tolerance and a reduced risk of developing T2D in older Swedish women [96]. Other studies showed that CMPF was increased in T2D and gestational diabetes [97,98,99,100,101]. These data suggest that plasma CMPF concentrations may reflect two different outcomes: as a modulator of β-cell dysfunction leading to T2D, and in populations without T2D, as a possible biomarker of fish intake [96]. Furthermore, the effect of CMPF could be dose-dependent, with moderate concentrations having a protective role, while higher doses may induce destruction of pancreatic β cells and progression to T2D. As the relationship between CMPF and T2D is not yet well established, further research is required.

In summary, the potential of FuFAs in liver disease remains unconfirmed due to the paucity of studies. However, the numerous biological properties of FuFAs, particularly against insulin resistance, could explain the protective effect of these fatty acids in MAFLD. Further mechanistic studies are indeed required to elucidate the underlying pathways to better understand the molecular mechanisms driving FuFAs’ effects on hepatic metabolism and insulin sensitivity. A metabolite of FuFAs, CMPF, might be of particular interest, as it may have insulin-sensitizing properties, which could account for this protective feature.

## 5. Branched Fatty Acid Esters of Hydroxy Fatty Acids (FAHFAs)

FAHFAs are valuable minor bioactive components found in both plant and animal-derived foods. Nut oils, grains, and teas are excellent plant sources [102]. Clementine, garlic, and pineapple are also rich in FAHFAs [103]. Caribou and moose meat, egg yolk, and beef have been identified as high or significant dietary sources (Table 6) [104]. A review on FAHFA diversity and content in various foods has been recently published [102]. To our knowledge, daily FAHFA consumption has never been estimated, but omnivores tend to have higher FAHFA levels than vegetarians/vegans [105].

### 5.1. FAHFAs Structure and Metabolism

**Figure 3:**
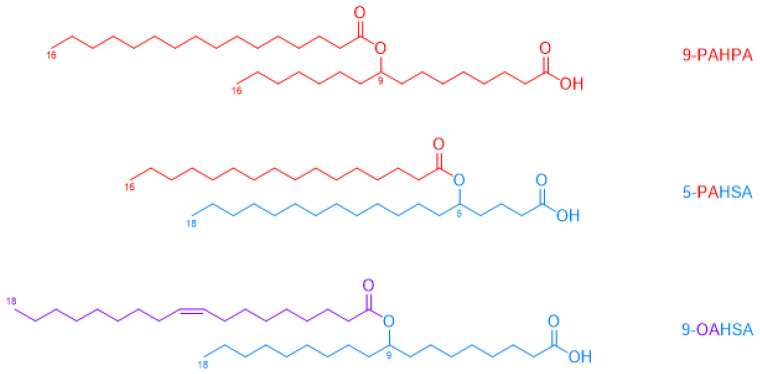
Structure of 3 FAHFAs: 9-PAHPA, 5-PAHSA, and 9-OAHSA. Red: Palmitic Acid. Blue: Stearic Acid. Purple: Oleic Acid.

FAHFAs belong to the estolide family, characterized by at least two fatty acids linked by an ester. As the name suggests, FAHFAs are a combination of a fatty acid (FA) (e.g., oleic acid, OA) esterified with a hydroxy fatty acid (HFA) (e.g., hydroxystearic acid, HSA), abbreviated as OAHSA. The ester position (branching carbon) defines a regioisomer (e.g., 9-OAHSA, as seen in Figure 3) [106]. Two superfamilies of FAHFAs can be distinguished based on the position of the estolide bond: in-chain branched FAHFAs and linear (ω-hydroxylated) FAHFAs. Sixteen FAHFA families consisting of several regioisomers were first discovered by Professor Kahn’s team in 2014 [107]. Nearly 50 families of these fatty acids have since been identified, with PAHSA being the most studied. The diversity of possible HFAs and FAs generates many unique structures in the FAHFA families. Branched FAHFAs can be derived from saturated lipids (e.g., 5-PAHSA palmitic acid-5-hydroxystearic acid), monounsaturated lipids (e.g., 9-OAHSA oleic acid-9-hydroxystearic acid), or polyunsaturated lipids (e.g., 13-DHAHLA docosahexaenoic acid-13-hydroxy-linoleic acid) [108].

FAHFAs can be synthesized endogenously, though the in vivo biosynthetic pathway remains poorly understood. It is suspected that an unidentified enzyme from the acyltransferase family catalyzes the esterification of a hydroxy fatty acid with an acyl-CoA fatty acid [107,109]. The presence of different FAHFA regioisomers in various organs suggests that specific acyltransferases may exist in different cell types, though they have yet to be identified. Further research is needed to elucidate the FAHFA degradation pathway. Several hydrolases, such as carboxylester lipase (CEL), carboxylesterase 3, androgen-induced gene 1 protein (AIG1), and androgen-dependent TFPI-regulating protein (ADTRP), have been proposed as potential estolide catalyzers [110,111].

In humans, FAHFAs are found in the blood and various tissues, including adipose tissue, liver, muscle, pancreas, kidney, and lung. Yore et al. [107] and Kuda et al. [112] reported total PAHSA levels of approximately 10 nM in human and mouse serum. In tissues, the highest PAHSA levels were found in white and brown adipose tissues (200 pM), while lower levels were found in the liver (50 pM) and pancreas (20 pM). Interestingly, breast milk from obese and lean pregnant women contained PAHSA regioisomers, particularly 5-PAHSA, as well as other FAHFA families [113]. It is noteworthy that physiological and pathophysiological changes such as fasting, refeeding, insulin resistance, and obesity regulate PAHSA levels [107,114,115].

### 5.2. Benefits of FAHFAs on Liver Health

Since their discovery in 2014, FAHFAs have shown beneficial effects on human health, with particular relevance to their potential role in the treatment of T2D [107]. They reduce inflammation [107,112,116,117] and possess antioxidant properties [118]. As previously stated in the introduction to this review, MAFLD is closely linked to T2D and insulin resistance. Disruption of lipid homeostasis can result in oxidative stress and inflammation, suggesting that FAHFAs might be beneficial in the context of hepatic steatosis. However, only a limited number of studies have investigated the impact of FAHFAs on MAFLD (Table 7).

Findings on the effects of FAHFAs on hepatic steatosis are conflicting. We recently demonstrated that long-term high intake of 9-PAHPA and 9-OAHPA improved peripheral insulin sensitivity in healthy mice [119] and that these two FAHFAs induce a shift in skeletal muscle toward a more oxidative contractile phenotype, potentially explaining their insulin-sensitizing properties [120]. Thus, sarcopenia, characterized by insulin resistance, impaired muscle regeneration, and its close association with MAFLD [121], could serve as a therapeutic target for FAHFAs. However, FAHFAs supplementation may lead to liver steatosis and fibrosis in some patients [119]. One hypothesis suggests that excessive insulin sensitization in a healthy liver may trigger de novo lipogenesis, leading to steatosis and fibrosis. Surprisingly, genes involved in lipogenesis and inflammation were not significantly different from controls, and FAHFAs had no effect on liver mitochondrial activity and oxidative stress. The cause of liver fibrosis in these mice remains unclear. Conversely, supplementation with 9-PAHPA and 9-OAHPA did not affect liver steatosis in HFHS diet-fed, diet-induced obese (DIO), or db/db mice [122,123]. On the other hand, Wang et al. reported that administering 5-PAHSA for one month led to hepatic steatosis in db/db mice. They also observed elevated serum levels of interleukin-1α, *C*-reactive protein, and tumor necrosis factor-α (TNF-α), indicating potential progression toward liver inflammation [124]. They hypothesized that higher hyperglycemia in the db/db mouse model was responsible for these results. This was confirmed in vitro in human HepG2 cells, where elevated glucose levels masked the beneficial effects of 5-PAHSA on decreased lipogenesis, increased fatty acid oxidation, and inhibition of the nuclear factor-κB pathway. In contrast, Moreira et al. found that 9-PAHSA supplementation prevented lipid accumulation in HepG2 and primary murine hepatocytes exposed to oleic acid (OA) [125].

Regarding hepatic glucose metabolism, Zhou et al. demonstrated that chronic administration of 9-PAHSA improved hepatic insulin sensitivity in chow and HFD-fed mice, reducing endogenous glucose production. In mouse hepatocytes, 9-PAHSA increased insulin signaling and glycogen synthesis while decreasing gluconeogenesis by inhibiting the cAMP signaling pathway, thus reducing G6Pase activity. Additionally, 9-PAHSA administration indirectly attenuated white adipose tissue lipolysis, decreasing hepatic glucose production [126].

In terms of liver inflammation, FAHFAs have shown promising effects. Situmorang et al. reported that in rat hepatocytes with LPS-induced inflammation, 9-PAHSA inhibited nuclear translocation of p65, reduced the expression of the proinflammatory cytokines TNF-α and IL-6, as well as connective tissue growth factor (CTGF), and reduced cell migration. These observations suggest a protective role of 9-PAHSA against liver fibrosis [127]. Another study highlighted the anti-inflammatory effects of 9-OAHSA, which decreased lipoapoptosis in rat hepatocytes exposed to palmitic acid and in Syrian hamsters fed a diet high in fat, cholesterol, and fructose. In rat hepatocytes, 9-OAHSA reduced mitochondrial ROS production and stabilized mitochondrial membrane potential. It also improved the expression of protein kinase Cδ, associated with cellular function. In Syrian hamsters, 9-OAHSA reduced apoptosis, as evidenced by decreased cleaved caspase 3 expression and phosphorylation of extracellular signal-regulated kinase (ERK) and Jun *N*-terminal kinase (JNK) [128]. Moreira et al. showed that 9-PAHSA increases cell viability of HepG2 and primary mouse hepatocytes in a dose-dependent manner following the addition of OA, suggesting that 9-PAHSA mitigates OA-associated lipotoxicity in these cells [125].

In the context of obesity, FAHFAs have shown beneficial effects. Wang et al. demonstrated that 9-PAHSA treatment of 3T3-L1 adipocytes increased the expression of brown fat-specific genes, such as uncoupled protein 1 (UCP1), which is crucial for thermogenesis. This anti-obesogenic effect was also observed in the white adipose tissue of wild-type and ob/ob mice [129]. Browning of white adipocytes induced by 9-PAHSA and 9-PAHPA was recently confirmed in the human multipotent adipose-derived stem cell model [130]. FAHFAs also exhibit anti-diabetic properties. PAHSAs improve basal serum glucose levels, enhance insulin secretion directly or indirectly via glucagon-like peptide-1 secretion, and increase glucose transport [107]. Benlebna et al. investigated the effects of 9-PAHPA and 9-OAHPA on healthy and DIO C57BL/6J mice [119,122]. They observed a decrease in basal metabolism and a lower respiratory exchange ratio (RER), indicating a shift towards carbohydrate use over fatty acids. This phenotype is associated with diabetes and insulin resistance. Both FAHFAs increased insulin sensitivity (insulin tolerance test) but did not affect glucose tolerance (oral glucose tolerance test) or hepatic insulin resistance (HOMA-IR). Rychtrmoc et al. documented that C57BL/6J mice fed a normal diet with either 9-PAHSA or 9-OAHSA had lower glycemia in the 9-PAHSA group compared to controls and the 9-OAHSA group, suggesting isoform-dependent hypoglycemic properties of FAHFAs [131]. The insulin-sensitizing properties of FAHFAs may be due to a shift towards an oxidative contractile phenotype in skeletal muscle. Long-term supplementation with 9-PAHPA and 9-OAHPA in healthy C57BL/6J mice increased the proportion of contractile IIa and IIx fibers in the tibialis muscle, suggesting a more oxidative muscle metabolism [105,120]. When used as a curative treatment—administered eight weeks after initiating a high-fat, high-sugar (HFHS) diet—9-PAHPA improved insulin sensitivity in both HFHS-fed and control db/db mice. However, it did not affect hyperglycemia or weight gain [123]. Unlike preventive treatment, curative treatment with 9-PAHPA and 9-OAHPA had no effect on basal metabolism and energy expenditure in these obesity models. Regarding the FAHFAs content in various foods [102], the doses of FAHFAs tested in animal studies are at least 10 times higher than those provided by the human diet.

**Table 7:** Overview of FAHFA Studies on the Impact of MAFLD-Related Parameters.

Author, Year	FAHFAs	Experimental Models	Dose & Duration	Conclusion
Wang et al., 2018 [129]	9-PAHSA	Mouse Model (obesogenic mice, oral gavage) In vitro model 3T3-L1	50 mg/kg/day (4 weeks) 5/10/20/30 μM (8 days)	Increased browning of white adipose tissue Increased browning of 3T3-L1 adipocytes
Wang et al., 2019 [124]	5-PAHSA	Mouse model (obesogenic mice, oral gavage) In vitro model (HepG2, 3T3-L1)	50–150 mg/kg/day (1 month) 20 μM (2 days)	No effect on body weight, glycemia, and insulin levels Increased liver steatosis and inflammation Improvement in glucose tolerance Significant decrease in insulin resistance
Zhou et al., 2019 [126]	5-PAHSA 9-PAHSA	Mouse model (obesogenic mice, IV infusion)	30 mg/kg/day (up to 29 weeks)	Decreased glycemia and white adipose tissue lipolysis Increased hepatic and systemic insulin sensitivity
Schultz Moreira et al., 2020 [125]	9-PAHSA	In vitro model (HepG2 and primary murine hepatocytes)	5/10/20/40 μM (6 h)	Improvement in mitochondrial dysfunction Reduction in lipid accumulation
Benlebna et al., 2020 [119]	9-PAHPA 9-OAHPA	Mouse model (obesogenic mice, dietary intake)	15 mg/kg/day (12 weeks)	No effect on body weight, glucose tolerance, liver steatosis, and oxidative stress Increase in basal metabolism Improvement in insulin sensitivity
Benlebna et al., 2021 [122]	9-PAHPA 9-OAHPA	Mouse model (healthy mice, dietary intake)	15 mg/kg/day (12 weeks)	No effect on obesity, hyperlipidemia, glucose intolerance, liver steatosis, and oxidative stress Amelioration of peripheral insulin resistance Thermogenic phenotype in white adipose tissue induction
Bonafos et al., 2023 [123]	9-PAHPA	Mouse model (obesogenic mice, dietary intake)	15 mg/kg/day (8 weeks)	No effect on body weight, energy expenditure, hyperglycemia, liver steatosis, and oxidative stress Improvement in insulin sensitivity
Situmorang et al., 2023 [127]	9-POHSA	In vitro model (rat hepatocytes)	2.5/5/10 μM (24 h)	Decreased IL6, TNF-α, and connective tissue growth factor (CTGF) expression
Loh et al., 2023 [128]	9-OHSA	Syrian hamster model (obesogenic model, continuous delivery via mini-osmotic pump implanted subcutaneously) In vitro models (rat hepatocytes)	ND (42 days) 10/20/40 μM (24 h)	Decreased lipoapoptosis and dyslipidemia Decreased apoptosis and oxidative stress Improvement in protein kinase Cδ
Colson et al., 2023 [130]	9-PAHPA 9-PAHSA	In vitro model (human multipotent adipose-derived stem cell)	1/3/10 Μm (4 days)	Increased browning of white adipocytes

Despite the limited number of studies on FAHFAs in liver disease, promising beneficial effects have been demonstrated. However, these effects remain speculative, as no human studies have yet been conducted to confirm these observations. In particular, 9-PAHSA has shown positive results in improving hepatic insulin resistance and reducing glucose production. Additionally, the anti-inflammatory properties of FAHFAs have been documented in the liver. FAHFAs also offer potential benefits against obesity and diabetes, which are key risk factors in the pathogenesis of MAFLD.

## 6. Conclusions

Bioactive lipids play significant roles in liver health and disease (see Graphical Abstract). These molecules, which include *n*-3 fatty acids, furan fatty acids (FuFAs), and the recently discovered fatty acid esters of hydroxy fatty acids (FAHFAs), influence liver function and pathology.

*n*-3 FA are essential fatty acids known for their anti-inflammatory, anti-thrombotic, and anti-arrhythmic properties. The impact of these polyunsaturated fatty acids on liver diseases has been extensively studied. PUFAs promote fatty acid oxidation and inhibit lipogenesis, decrease inflammation and improve insulin sensitivity, which are critical factors in the prevention and management of various liver diseases. FuFAs are dietary fatty acids characterized by a furan ring. The antioxidant and anti-inflammatory properties of FuFAs, as described in the literature, suggest their potential as therapeutic agents for various liver diseases, including MAFLD. While direct evidence of FuFAs’ impact on hepatic steatosis is limited, they appear to reduce liver fat accumulation through their insulin-sensitizing effects. However, they seem to have no effect on oxidative stress. Further investigation is needed to confirm these findings. CMPF, a major endogenous human metabolite of FuFAs, has been implicated in this process, with several studies documenting its beneficial effect on liver steatosis. However, further research on the effect of CMPFs on liver steatosis is needed to draw firmer conclusions. FAHFAs are a novel class of bioactive lipids with anti-diabetic and anti-inflammatory effects. By improving insulin sensitivity and reducing inflammation, some FAHFAs may help prevent the development of MAFLD and progression to MASH. The role of FAHFAs in hepatic lipid accumulation remains uncertain, with inconsistent findings reported. It is important to note that research investigating the impact of FAHFAs/FuFAs on MAFLD is limited, primarily conducted in animal models and cell cultures. Current evidence is based solely on preclinical models.

In addition, these are not single, well-defined compounds, and significantly more research is needed to identify which specific compounds hold the greatest therapeutic potential. To date, while the evidence supporting the efficacy of *n*-3 fatty acids in MAFLD is robust, the data for FuFAs and FAHFAs remain relatively weak and inconclusive. Continued preclinical research, including mechanistic studies, is essential to fully exploit their therapeutic potential in liver disease. Future clinical trials are necessary to understand the mechanisms of action of these bioactive lipids and to establish their safety and efficacy in humans. Finally, the study of the biological properties and potential health effects of FuFAs and FAHFAs may offer an opportunity to diversify the sources of bioactive lipids and exploit their complementary benefits. As highlighted earlier, dietary modifications play a key role in preventing the onset and progression of MAFLD. Additionally, treatment strategies could include biofortified foods with enhanced omega-3 PUFAs, FuFAs, or FAHFAs contents, along with the use of nutritional supplements to aid in the management of MAFLD.

## Figures and Tables

**Table 1:** Overview of omega-3 sources and their concentrations [18].

Source	Concentration (mg/g)
Cod liver	
EPA	30
DHA	40
Fish	
EPA	3–30
DHA	3–33
Vegetable oil	
ALA	75–530

According to https://ciqual.anses.fr/ accessed on 30 January 2025.

**Table 2:** Overview table of included meta-analyses on the impact of *n*-3 fatty acids on MAFLD-related parameters.

Meta-Analysis (Author, Year)	No. of Studies	No. of Participants	Type of PUFA	Age (Year)	Dose (mg/day) & Duration	Outcomes Assessed	Conclusion (Effect on MAFLD-Related Parameters)
Moore et al., 2024 [47]	6	362 (210 adults–152 children/adolescents)	Plant-based omega-3 PUFAs	ND	ND (2–12 weeks)	Liver enzymes, glycemic control, blood lipid levels, body composition	Significant decrease in ALT (*p* = 0.02; 95% Confidence Interval (CI): −14.7, −1.38; *I*^2^ = 48.62), TAG (*p* = 0.01; 95% CI: −76.93, −12.08; *I*^2^ = 69.93%), and body composition factors: BMI (*p* < 0.001; 95% CI: −2.99, 0.68; *I*^2^ = 76.37%), waist circumference (WC) (*p* < 0.001; 95% CI: −4.92, −1.50; *I*^2^ = 20.32%, body weight (BW) (*p* < 0.01; 95% CI: −8.33, −1.04; *I*^2^ = 0.00%) No effect on AST, GGT, blood glucose, and HOMA-IR Non-significant decrease in LDL-C
Musazadeh et al., 2023 [48]	8	6561	Marine-based omega-3 PUFAs	40	250–5000 (8–72 weeks)	Liver fat content, liver enzymes	Significant decrease in AST (*p* < 0.001; 95% CI: −8.61, −4.84; *I*^2^ = 23.4%), ALT (*p* < 0.000; 95% CI: −5.93, −1.53; *I*^2^ = 0%), GGT (*p* < 0.002; 95% CI: −6.85, −1.55; *I*^2^ = 47.7%) Non-significant decrease in liver fat content
Lee et al., 2020 [49]	22	1366	Marine-based omega-3 PUFAs	ND	250–5000 (8–72 weeks)	Liver fat content, liver enzymes, blood lipid levels, glycemic control, body composition	Significant improvement in liver fat content (*p* < 0.01; 95% CI: 1.09, 2.13; *I*^2^ = 49%), levels of TAG (*p* = 0.0001; 95% CI: −40.81, −16.33; *I*^2^ = 64%), TC (*p* = 0.0002; 95% CI: −14.86, −0.79; *I*^2^ = 64%), HDL-C (*p* < 0.0001; 95% CI: 1.38, 5.73; *I*^2^ = 70%), and BMI (*p* = 0.04; 95% CI: −0.84, −0.08; *I*^2^ = 44%)
Musa-Veloso et al., 2018 [50]	18	1132	Long-chain omega-3 PUFAs (EPA/DHA)	41	1000–4000 (8–96 weeks)	Liver fat content, liver enzymes, blood lipid levels, glycemic control, body composition	Significant decrease in ALT (*p* = 0.046; 95% CI: −9.18, −0.08; *I*^2^ (ND)), GGT (*p* < 0.05; 95% CI: −8.92, −1.40; *I*^2^ (ND)), liver fat content (*p* = 0.021; 95% CI: −9.58, −0.79; *I*^2^ (ND)), TC (*p* = 0.035; 95% CI: −15.8, −0.60; *I*^2^ (ND)), TAG (*p* < 0.001; 95% CI: −36.7, −12.9; *I*^2^ (ND)), LDL-C (*p* = 0.001; 95% CI: −11.4, −3.1; *I*^2^ (ND)), HOMA-IR (*p* = 0.008; 95% CI: −0.93, −0.14; *I*^2^ (ND)) and BMI (*p* = 0.005; 95% CI: −1.44, −0.26; *I*^2^ (ND)) Significant increase in HDL-C (*p* < 0.001; 95% CI: 1.5, 4.8; *I*^2^ (ND)) Non-significant decrease in AST No effect on fasting blood glucose, adiponectin, BW, and WC
Yan et al., 2018 [51]	18	1424	Marine-based omega-3 PUFAs	43	250–1000 (12–96 weeks)	Liver fat, liver enzymes, blood lipid levels, glycemic control, body composition	Significant decrease in ALT (*p* < 0.001; 95% CI: −0.88, −0.11; *I*^2^ = 86.4%), AST (*p* < 0.001; 95% CI: −1.04, −0.05; *I*^2^ = 91.2%), TAG (*p* < 0.01; 95% CI: −0.76, −0.19; *I*^2^ = 79.6%), and liver fat (*p* = 0.018; 95% CI: 1.23, 1.98; *I*^2^ = 60.7%) Non-significant effect on TC, HDL-C, LDL-C, insulin, BMI, and WC
Guo et al., 2018 [56]	11	536	Marine-based omega-3 PUFAs	47	450–5000 (8–72 weeks)	Liver fat content, liver enzymes, blood lipid levels, glycemic control	Significant decrease in ALT (*p* < 0.001; 95% CI: −9.98, −5.08, 1.98; *I*^2^ = 0%), AST (*p* = 0.002; 95% CI: −11.67, −2.52, 1.98; *I*^2^ = 83.4%), liver fat (*p* = 0.051; 95% CI: −10.24, 0.02; *I*^2^ = 72.1%), TAG (*p* < 0.001; 95% CI: −49.15, −23.18; *I*^2^ = 51%) Non-significant decrease in fasting glucose
Yu et al., 2017 [52]	13	668	Marine-based omega-3 PUFAs	40	1200–4500 (12–96 weeks)	Liver enzymes, blood lipid levels, glycemic control	Significant decrease in AST (*p* < 0.0001; 95% CI: −12.65, 2.51; *I*^2^ = 84%) and TAG (*p* = 0.04; 95% CI: −48.22, −9.91; *I*^2^ = 57%) Significant increase in HDL-C (*p* = 0.009; 95% CI: 1.59, 8.03; *I*^2^ = 65%) Non-significant decrease in ALT, GGT, and fasting glucose
He et al., 2016 [53]	7	442	Marine-based omega-3 PUFAs	49	2000–6400 (24–96 weeks)	Liver enzymes, blood lipid levels, glycemic control, liver fat content and fibrosis	Significant decrease in AST (*p* = 0.002; 95% CI: −17.71, 3.92; *I*^2^ = 76%), TC (*p* = 0.04; 95% CI: −21.44, −5.38; *I*^2^ = 56%), LDL-C (*p* = 0.010; 95% CI: −14.26, −0.00; *I*^2^ = 70%) Trend towards an increase in HDL-C Trend towards a decrease in ALT, TAG, and GGT No effect on liver fibrosis
Lu et al., 2016 [54]	10	577	Marine-based omega-3 PUFAs	ND	830–9000 (8–96 weeks)	Liver fat content, liver enzymes, blood lipid levels	Significant decrease in liver fat (*p* = 0.01; 95% CI: 1.31, 9.89; *I*^2^ = 51%), GGT (*p* = 0.002; 95% CI: −14.80, −3.24; *I*^2^ = 0%), and TAG (*p* = 0.0001; 95% CI: −53.90, 17.90; *I*^2^ = 73%) Trend towards decrease in ALT, AST, TC, and LDL-C Significant increase in HDL-C (*p* = 0.05; 95% CI: 0.03, 11; *I*^2^ = 80%)
Parker et al., 2012 [55]	9	355	Marine-based omega-3 PUFAs	ND	830–13,700 (8–48 weeks)	Liver fat, liver enzymes	Significant decrease in liver fat (*p* < 0.001; 95% CI: −0.48, −1.72; *I*^2^ = 66.12%), AST (*p* < 0.001; 95% CI: −0.16, −0.60; *I*^2^ = 91.62%), and ALT fat (*p* = 0.001; 95% CI: −0.06, −0.44; *I*^2^ = 88.32%)

ND: Non Determined.

**Table 3:** Overview of FuFA sources and their concentrations [18].

Source	Concentration (μg/g)
Fish	4–250
Shellfish [71]	5–200
Dairy products	50 to 500
Vegetable oils	2 to 400
Vegetables/fruits/rice/wheat	1 to 350 (dry weight)

**Table 4:** Nomenclature according to Vetter [77].

Acronym	Nomenclature
9D3	9-(3,4-dimethy-5-propyl-furan-2-yl) nonanoic acid
9M5	9-(3-methy-5-pentyl-furan-2-yl) nonanoic acid
9D5	9-(3,4-dimethy-5-pentyl-furan-2-yl) nonanoic acid
11D3	11-(3,4-dimethy-5-propyl-furan-2-yl) undecanoic acid
11M5	11-(3-methy-5-pentyl-furan-2-yl) undecanoic acid
11D5	11-(3,4-dimethy-5-pentyl-furan-2-yl) undecanoic acid
13M5	13-(3-methy-5-pentyl-furan-2-yl) tridecanoic acid
13D5	13-(3,4-dimethy-5-pentyl-furan-2-yl) tridecanoic acid

**Table 5:** Overview of studies on the impact of FuFAs on MAFLD-related parameters.

Author, Year	FuFAs	Experimental Model	Dose & Duration	Conclusion
Wakimoto et al., 2011 [88]	11D5	Rat model of adjuvant-induced arthritis (oral gavage)	1 to 10 mg/kg/day (day 10 to day 15)	Dose dependent effect 10 mg/kg of F6 ethyl ester showed 74% suppression of paw swellin
Khan et al., 2018 [87]	11D5	Human neutrophil	0.05 to 5 µg/0.1 mL (120–240 min)	Induced NETosis in human neutrophils
Lauvai et al., 2019 [86]	9M5	3T3-L1 preadipocytes	10 µM (7 days)	Increased protein expression of PPARγ, C/EBPα, FABP4, and adiponectin
Dore et al., 2023 [89]	9M5	DIO mice (in diet)	40 mg and 110 mg/kg/day (3 months)	Reduced liver steatosis, improved insulin sensitivity, decreased fat mass, restored normal energy expenditure and increased muscle mass

**Table 6:** Overview of FAHFAs sources and their concentrations.

Source	Concentration (ng/g)
Moose/Caribou [103]	50 × 10^3^
Vegetables/rice/wheat [105]	10 to 320
Meat [106]	3–8
